# The Story of the Insane from Year to Year

**Published:** 1895-08-31

**Authors:** 


### THE STORY OF THE INSANE FROM YEAR TO YEAR.

Eighth Series.

### GOVAN ASYLUM.

This is a small parochial asylum situate at Govan, near Glasgow. It contained 263 patients on the first day of 1894, and the number had increased by two only at the end of the year. There were 74 admissions, 27 recoveries, and 24 deaths. The recoveries were 36·5 per cent. on the admissions, and the deaths were 8·0 per cent. on the average number resident. Twelve of the deaths were due to brain diseases and only 2 to consumption. Hereditary tendency caused the insanity in 7 of the admissions, drink in 13, epilepsy in 6, and in 34 no cause was ascertained. Dr. Watson resigned the office of medical superintendent on being appointed to the new asylum at Hawkhead, and Dr. Richard now reigns in his place. The report is a very short one. The Committee of Visitors do not publish any remarks, and no table showing the cost per head is given.

### THE LAWN HOSPITAL FOR THE INSANE AT LINCOLN.

At the end of the year this hospital contained 69 patients. There were 21 admissions, 10 recoveries, 3 discharged relieved, and 3 deaths. The recoveries stand at 50 per cent. on the admissions, and the deaths 4 per cent. on the average number resident. The rcoveries are therefore very high and the death rate very low. We notice that there was no serious accident of any kind, and that neither seclusion nor restraint had been employed during the year. Many improvements have been carried out, and the hospital is now in possession of a handsome recreation room. The governors say they "highly appreciate the efforts which are so successfully made by the medical superintendent to relieve the monotony of the lives of the patients by concerts, dances, theatrical performances and other innocent amusements; and they believe the high percentage of recoveries has been largely owing to the judicious blending of amusements with the control incidental to an institution for dealing with the insane."

### SUFFOLK COUNTY ASYLUM.

In this asylum the numbers increased from 496 to 551. There were, including transfers, 211 admissions, 77 recoveries, and 66 deaths. The percentage of recoveries stands at 45·29 on the admissions, and that of the deaths at 12·66 on the average number resident. Both of these are high.

Of the deaths, 19 were caused by cerebral and spinal diseases, 13 by consumption, 13 by dysentery, and one by enteric fever. Indeed, Dr. Eager must have had his hands full, for we notice there were 83 cases of dysentery, and this number included two of the staff. This bears out what we have stated in these notices as to the much greater frequency with which dysentery attacks the insane than the sane. Here we had this disease attacking about one in seven of the patients, and only one in 35 of the staff. Dr. Eager's report is a long one, running to nearly 30 pages, and deals in a very exhaustive way with the condition of the asylum, more especially as regards the water supply. No doubt many parts of an asylum so old as the Suffolk County one must need remodelling and improving, but there would seem to be an unusual objection on the part of the ratepayers to spend money for these purposes. Dr. Eager says this is owing "to their incapacity to grasp the benefits to be derived," and he thinks this "should not absolve their representatives from performing their duties to them and to those in their care." The average weekly cost per head is 9s. 6d.

### BELFAST DISTRICT ASYLUM.

The limits of accommodation are given at 550 beds, but we find that the asylum contained 706 patients on January 1st, 1894, and this number had risen to 750 at the end of the year. It is curious to notice how often the population of Irish asylums is in excess of the proper accommodation, and we should like to learn who is to blame for such a grave mistake. In this asylum the admissions reached 1215, the recoveries 100, the discharged (relieved) 20, and the deaths 45. The recoveries were 46·5 per cent. of the admissions, and the deaths were only 6·2 per cent. on the average number resident. Twenty-seven of the deaths were from cerebral and spinal diseases (one of these was general paralysis), three were from consumption, and two from suicide. Domestic trouble and adverse circumstances are the assigned causes of insanity in 17 cases, religion in 16, intemperance in drink in 12, and hereditary influence in 38. With the object of providing proper accommodation for the insane poor an estate of 300 acres has been bought, and in the meantime the mansion on the estate is to be altered so as to take in 75 male patients. In spite of the overcrowding the health of the patients has been good, and the death-rate exceptionally low. The average cost per head per annum was £24 15s. 2d.

### BERKS COUNTY ASYLUM AT MOULSFORD.

On January 1st this asylum contained 545 patients, and on the last day of the year the numbers had risen to 554. The admissions were 117, the recoveries 43, and the deaths 50. The recoveries stand at 36·7 per cent. on the admissions, but transfers from other asylums are included in this calculation. The deaths are a fraction over 9 per cent. on the average number resident. Of the deaths 10 were from cerebral or spinal diseases, 7 from consumption, 5 from enteric fever, and 1 from dysentery. Hereditary predisposition, either alone or in conjunction with other causes, is given as the cause of the insanity in 34 of the admissions; domestic trouble and adverse circumstances were the cause in 5, while ntemperance in drink is credited with causing the disease in only 4 cases. We notice, however, that "previous attacks" are the cause in 11, and that 13 were "unknown." Dysentery and typhoid fever broke out during the year, and, as already stated, the former disease caused 1 death, and the latter 5. These diseases were confined to the patients. This peculiarity has been noticed in many other asylums, and is not easy to account for. Of course the patients are ten times as numerous as the staff, and therefore would suffer in higher numbers; but the asylum form of dysentery has been again and again known to break out among the patients, attacking ten or twenty or more, while the attendants have entirely escaped. And yet, as Dr. Murdoch points out, "staff and patients live under exactly the same sanitary conditions, use the same water, and practically have the same food." The whole sanitary system is being overhauled, and where defective will be put to rights. An infectious hospital capable of holding 12 patients has been built at a cost of £2,879, so that it should be a model of its kind. The committee "desire to express their continued high appreciation of the services of Dr. Murdoch." The cost per head per week is 7s. 9d.

